# Evaluating brain modularity benefits of an acting intervention: a discriminant-analysis framework

**DOI:** 10.3389/fnhum.2023.1114804

**Published:** 2023-05-04

**Authors:** Aishwarya Rajesh, Richard Betzel, Ana M. Daugherty, Tony Noice, Helga Noice, Pauline L. Baniqued, Michelle W. Voss, Arthur F. Kramer

**Affiliations:** ^1^Department of Psychology, University of Illinois at Urbana-Champaign, Champaign, IL, United States; ^2^Department of Psychological and Brain Sciences, Indiana University, Bloomington, IN, United States; ^3^Department of Psychology, Wayne State University, Detroit, MI, United States; ^4^Department of Theater and Dance, Elmhurst University, Elmhurst, IL, United States; ^5^USC Center for Affective Neuroscience, Development, Learning, and Education, University of Southern California, Los Angeles, CA, United States; ^6^Department of Psychological and Brain Sciences, The University of Iowa, Iowa City, IA, United States

**Keywords:** acting, executive function, modularity, aging, brain

## Abstract

**Purpose:**

Aging is associated with a reduction in brain modularity as well as aspects of executive function, namely, updating, shifting, and inhibition. Previous research has suggested that the aging brain exhibits plasticity. Further, it has been hypothesized that broad-based intervention models may be more effective in eliciting overall gains in executive function than interventions targeted at specific executive skills (e.g., computer-based training). To this end, we designed a 4-week theater-based acting intervention in older adults within an RCT framework. We hypothesized that older adults would show improvements in brain modularity and aspects of executive function, ascribed to the acting intervention.

**Materials and methods:**

The participants were 179 adults from the community, aged 60–89 years and on average, college educated. They completed a battery of executive function tasks and resting state functional MRI scans to measure brain network modularity pre- and post-intervention. Participants in the active intervention group (*n* = 93) enacted scenes with a partner that involved executive function, whereas the active control group (*n* = 86) learned about the history and styles of acting. Both groups met two times/week for 75-min for 4 weeks. A mixed model was used to evaluate intervention effects related to brain modularity. Discriminant-analysis was used to determine the role of seven executive functioning tasks in discriminating the two groups. These tasks indexed subdomains of updating, switching, and inhibition. Discriminant tasks were subject to a logistic regression analysis to determine how post-intervention executive function performance interacted with changes in modularity to predict group membership.

**Results:**

We noted an increase in brain modularity in the acting group, relative to pre-intervention and controls. Performance on updating tasks were representative of the intervention group. However, post-intervention performance on updating did not interact with the observed increase in brain modularity to distinguish groups.

**Conclusion:**

An acting intervention can facilitate improvements in modularity and updating, both of which are sensitive to aging and may confer benefits to daily functioning and the ability to learn.

## Introduction

Aging is associated with alterations in macroscale brain topography and topology which may be related to cognitive decline (Iordan et al., 2018). Older adults show reduced modular brain organization, consistent with the theory of age-related functional de-differentiation (Betzel et al., 2014; Cao et al., 2014; Chan et al., 2014; Geerligs et al., 2015; Sala-Llonch et al., 2015; Baniqued et al., 2018; Iordan et al., 2018). Furthermore, aging is associated with a decline in higher-order cognitive abilities known as executive functions—these functions are critical to both simple and complex tasks of daily living, including managing medications and driving (Marshall et al., 2011; Park et al., 2014; Maldonado et al., 2020). Thus, there is a need to identify preventative interventions that might optimize brain organization and protect cognitive abilities into advanced age. This study examined whether an intervention paradigm based on theater acting would facilitate an increase in modularity and executive function in older adults.

Recent advances in gerontological research have shown that lifestyle factors can shape individual neural cognitive aging trajectories. The potential for modifiable lifestyle factors to reduce age-related cognitive decline has motivated the pursuit of lifestyle-informed interventions. Such interventions are situated within enriched environments, for example, exercise (e.g., Voss et al., 2013; Baniqued et al., 2018) and theater acting (e.g., Noice and Noice, 2013; Banducci et al., 2017). They are not targeted at enhancing any single cognitive skill. Rather, they are targeted to a diversity of inter-related skills, facilitating improvements in multiple cognitive domains (e.g., Wilson et al., 2002; Wang et al., 2013; Baniqued et al., 2018). These broad-based interventions stand in contrast to performance-adaptive computerized cognitive training that purportedly tap into specific aspects of executive functions, but do not show transfer to general cognitive function (e.g., Ball et al., 2002; Dahlin et al., 2008; Wilkinson and Yang, 2012; Karbach and Verhaeghen, 2014; Grönholm-Nyman et al., 2017).

Assimilating these perspectives, it is evident that broad-based interventions are more likely to demonstrate global gains in executive functions. Preliminary evidence also suggests that baseline brain modularity is predictive of improvements in cognitive performance ascribed to such interventions (Baniqued et al., 2018; Gallen and D’Esposito, 2019).

Acting is one such broad-based intervention strategy that embeds individuals in complex social contexts. A particular training model within acting is called “active experiencing.” Unlike exercise, active experiencing involves a conscious and combined recruitment of cognitive and related affective and physiological processes. Thus, it likely involves integration among multiple higher-order cognitive functions, particularly, aspects of executive function. Executive functions are cognitive operations that involve updating contents of working memory, cognitive flexibility, and inhibitory control (Miyake et al., 2000).

Working memory is engaged in active experiencing when actors repeat their performances several times in rehearsal without making an active effort to memorize the dialogues. These performances are continuously adjusted based on the “give-and-take” from other actors, while maintaining goal-relevant information in working memory. The repetition of performances ensures constant updating of working memory that is richly contextual and elaborate, including visuo-spatial awareness. By and large, this updating “style” allows the actor to gradually absorb the dialogue, leading to greater memory retention [(Noice and Noice, 2013); see also “deep processing” as mentioned in Craik and Lockhart (1972)]. Similarly, cognitive flexibility is fostered as actors adapt to different character roles, change and adopt new tactics that may seem more appropriate for obtaining a desired goal, and respond to feedback and “on-the-fly” changes in scripts or actors (Noice and Noice, 2013). Lastly, inhibitory control is exercised when the actor utters a script line. At this time, various parameters of the actor’s rendition that are representative of the script line must be reflexively encoded, including vocal inflections, posture, and affect. Concomitant to the actor’s goals of the present moment, they must actively suppress any non-verbal parameters that are not representative of the script line.

Preliminary results of active experiencing-based acting as an intervention strategy have been promising. For instance, a 4-week acting intervention in older adults produced improvements on a wide array of cognitive measures including word recall, working memory, problem-solving abilities, and participants’ perceived quality of life (Noice et al., 2004). These cognitive gains remained 4 months after training stopped. Using a similar intervention model but with a considerably larger sample of older adults, we showed control-adjusted gains in episodic recall that persisted up to 4 months post-intervention (Banducci et al., 2017). Remarkably, these findings illustrate that even relatively short-term interventions (i.e., 4-weeks) can facilitate enduring improvements in cognitive performance across multiple domains.

Although there is promising evidence of cognitive gains, the parallel changes in brain morphology associated with the acting intervention have not been examined. A particularly relevant brain measure in this regard is modularity (Newman and Girvan, 2004; Bullmore and Sporns, 2009; Rubinov and Sporns, 2010). Modularity evaluates the presence of modules, which consist of brain regions that are densely connected with each other but sparsely connected with others (Power et al., 2011). Greater modularity implies the presence of more distinct modules defined by greater coherency and greater segregation of functional networks. In this regard, modular organization may represent the basic “building blocks” of the brain that inform network development and function as well as support brain integrity. Furthermore, modularity has been shown to be particularly sensitive to context, such that subtle changes in cognitive functioning that might occur within very short time periods are well-represented at the level of modular organization (Stevens et al., 2012). Older adults tend to show reduced modular brain organization (Betzel et al., 2014; Cao et al., 2014; Chan et al., 2014; Geerligs et al., 2015). This observation falls in line with the de-differentiation hypothesis, which posits that separate cognitive abilities developed in childhood become more highly correlated with age, resulting in “de-differentiation” (Goh, 2011).

Apropos the cognitive training literature, modularity has been shown to be predictive of cognitive gains across diverse populations and intervention models (Baniqued et al., 2018; Gallen and D’Esposito, 2019). Emerging evidence suggests that modularity may parallel cognitive gains when the training emphasizes flexible learning of a higher-order cognitive function (Finc et al., 2020). The present research built upon these observations and examined brain-wide modularity benefits associated with acting training, in the same large-scale study as Banducci et al. (2017). Further, we sought to determine whether changes in brain modularity were associated with changes in executive function attributed to intervention effects.

To this end, we conducted a 4-week active experiencing-based acting intervention in older adults. Participants were administered a battery of cognitive tests and underwent MRI scans before they were assigned to either the active control (*n* = 86) or the acting-intervention condition (*n* = 93), depending on time of enrollment. The active controls attended an Understanding the Art of Acting (UAA) class, which was a course in theater appreciation. The acting-intervention group trained in Active Experiencing (AE). Specifically, participants in this group repeatedly performed short scenes with a partner while making effortful attempts to embody their character cognitively, affectively, and physiologically. Both groups were led by professional actors/ theater professors and met two times/week for 75-min for 4 weeks.

Within the randomized control framework employed in this study, we tested three hypotheses. First, we anticipated increased modularity in the intervention group and greater gains as compared to the control group, reflecting flexible higher-order learning and optimization of executive function (Finc et al., 2020). Second, we hypothesized that post-intervention scores on executive function tasks would clearly discriminate the acting group from the active control group, ascribed to the active experiencing training model. Tasks that were deemed discriminatory informed our final hypothesis. Specifically, our third hypothesis was that post-intervention performance on discriminant tasks would positively interact with changes in modularity to predict group membership to acting.

## Materials and methods

### Participants

This study was approved by the Institutional Review Board at University of Illinois at Urbana-Champaign. Participants in this study were 179 adults from the community, aged 60–89 years (*M* = 69.46, SD = 6.59; 62% female). On average, they had a college education (*M* = 16.80 years, SD = 3.48). Inclusion criteria included right-handedness, a score of at least 23 on the Mini Mental State Examination (MMSE; *M* = 28.69, SD = 1.39; Folstein et al., 1975), no contraindication to MRI, and written consent to participate. For the CONSORT flow diagram of participant recruitment, enrollment, and attrition, see Banducci et al. (2017). The 179 participants were pseudo-randomly assigned to one of two groups depending on time of enrollment. One group was an active control group that attended an Understanding the Art of Acting class (the “UAA” group; *n* = 86). The other group was the active-experiencing based intervention group (the “AE” group, *n* = 93) that attended an acting class. The two groups were similar in age (*t*_(176)_ = –0.79, *p* = 0.43), MMSE (*t*_(176)_ = 0.22, *p* = 0.82) and years of education (*t*_(176)_ = 0.86, *p* = 0.39), as well as representation of sex (χ^2^_(1)_ ≤ 0.15, *p* ≥ 0.70).

### Intervention

Both control and intervention groups met two times per week for 75-minute sessions. These sessions also included a 15-min coffee break to facilitate social interaction.

The active control was designed as a theater appreciation class and included talks, demonstrations and video clips of stage and film performances (“Understanding the Art of Acting” or UAA group). Participants learned about different styles of acting, in addition to the history of theater. The UAA-control condition was designed to rule out the possibility that learning about a popular art form like acting, along with the social interaction of being engaged in a class, are sufficient to produce the significant improvement in cognitive functioning observed in the previous theater interventions.

Participants in the AE group trained by performing acting exercises and short scenes with a partner (with large print scripts 1–3 pages in length). Emphasis was given to “becoming” the character through the process of proactively and continuously investigating their character’s motivations. Concomitantly, participants were encouraged to embody their character cognitively, emotionally, and physically [for a more detailed review, see Noice et al. (1999)]. All participants were in the same room during classes, and active feedback was provided to the acting partners.

### Neuroimaging protocol

#### Image acquisition and pre-processing

All images were collected on a Siemens Trio 3 Tesla full body magnet, using a 12-channel birdcage head coil. Functional blood oxygenation level–dependent (BOLD) images were acquired parallel to the anterior commissure–posterior commissure (AC-PC) line with a T2*-weighted echo-planar imaging sequence of 35 contiguous axial slices collected in ascending order [repetition time (TR) = 2,000 msec; echo time (TE) = 25 ms; BOLD volumes = 180; flip angle = 80°; field of view [FOV] = 220mm × 220 mm; voxel size = 3.4 mm × 3.4 mm × 4.0 mm]. During this 6-min resting state scan, participants were instructed to keep their eyes closed.

Structural images were acquired with a T1-weighted three-dimensional (3D) magnetization prepared rapid gradient echo imaging (MPRAGE) protocol of 192 contiguous sagittal slices collected in an ascending manner parallel to the AC-PC line (TR = 1,900 ms; TE = 2.32 ms; flip angle = 9°; FOV = 230 mm × 230 mm; voxel size = 0.9 mm × 0.9 mm × 0.9 mm).

Image-processing was carried out with FSL version 5.0.4 (Functional Magnetic Resonance Imaging of the Brain’s Software Library^1^), AFNI, and MATLAB (The MathWorks, Natick, MA).

Functional pre-processing steps were as follows. First, raw DICOM images were converted to NIfTI format using FreeSurfer’s mri_convert tool and reoriented to RPI orientation with FSL’s fslorient. FSL’s BET (Brain Extraction Technique) algorithm was then used to strip voxels containing non-brain tissue from the high-resolution T1 structural images. Next, echo-planar imaging (EPI) data were motion corrected using AFNI’s 3dvolreg function, which produced six parameters of head motion. The motion-corrected EPI data were spatially smoothed using a full width at half maximum 6.0 mm Gaussian kernel.

#### Resting state

Following the rigid-body motion correction and spatial smoothing procedures above, we sought to remove as much remaining structured noise as possible. We used single-subject independent components analysis (ICA) as an additional denoising procedure. Specifically, we used an automated ICA procedure called MELODIC (FSL), which generates a set of spatial independent component maps (ICs), and associated time-courses for each participant. The resulting components are thought to represent brain activity (signal component), and/or structured noise (noise component).

The gold standard for classification of components as signal or noise remains the visual inspection of the components (Griffanti et al., 2017). To this end, we followed guidelines from Kelly et al. (2010). A component was labeled as noise when it showed clear evidence of a spotty or diffuse distribution of signal particularly in peripheral areas, and when such signal covered more than a quarter of the brain. When unclear, we used abrupt changes in time course, high power in high frequencies (>50% power above 0.1 Hz), and concentration of signal in the superior sagittal sinus to validate our labeling of a component as noise. Alternatively, a component was labeled as signal when clusters of brain activity were clearly evident in gray-matter regions (Kelly et al., 2010). Components representing noise were regressed out from the data, thus allowing us to retain as much signal as possible, while removing as much structured noise as possible through this ICA-artifact removal process.

To further remove confounding signals, such as those due to cardiac pulse or low frequency scanner drift, the images corrected for noise were bandpass filtered for frequencies above 0.008 Hz and below 0.1 Hz, using AFNI’s 3dBandpass tool. This band-pass filter also was applied to the six motion parameters mentioned previously. The mean time series was then extracted from specific regions in deep white matter (retro-lenticular portion of the left internal capsule) and cerebrospinal fluid (left ventricle) and entered into the general linear model (GLM) as nuisance regressors.

The residual time series from this nuisance regression was then evaluated for any motion-contaminated volumes, given that motion-related artifacts can persist even after the extensive processing steps described above (Power et al., 2012). Briefly, for each participant, a temporal mask was generated for successive volumes with frame-wise (head) displacement that exceeded 0.5 mm and for successive volumes with changes in BOLD that exceeded a spatial standard deviation of 0.5% between them. These temporal masks were enhanced by also marking the frames 1 back and 2 forward from any marked frames to account for temporal smoothing of the BOLD data. The intersection of these two masks was used to create a final temporal mask for each participant, which eliminated their marked frames from further data analysis. This procedure affected three participants (one participant pre-intervention with five marked frames, two participants post-intervention with three and five marked frames). We did not account for within-volumes displacement. The resulting functional image was registered to MNI space before extracting timeseries for modularity analysis.

### Behavioral measures

Participants completed tasks that pre-dominantly characterize specific aspects of executive function (i.e., updating, shifting, inhibition), as well as generalized executive functions that tap into all three components and may overlap with other higher-order cognitive functions. Individuals who administered these tasks were blind to intervention assignment. The executive function tasks included the Spatial working Memory task, the 2-back task, Task Switching, the DKEFS Trail Making test, the Flanker task, the Digit Symbol Substitution task, and the Means-Ends Problem Solving task. On these tasks, we opted for performance measures that showed greatest variability across participants and across time. Within this context, when a task evaluated both reaction time and accuracy, we chose reaction time as the performance measure of interest. Reaction time on such tasks was computed only for accurate trials. These tasks and performance measures are described in greater detail in Table 1.

**TABLE 1:** Qualitative descriptions for tasks.

Task measure	Type (C = Computerized, W = Written)	Brief description	Measure used for present study
**Updating**			
Spatial Working Memory (Banducci et al., 2017)	C	It is administered at variable difficulty memory loads of two, three and four target dots. These dots are randomly arranged and are visible for a very short duration. After this presentation of dots, a red dot appears. Participants indicate whether the red dot appears in the same location as one of the black dots that was just presented.	Reaction time for the highest cognitive load condition was used (i.e., 4-dot condition).
2-Back (Gajewski et al., 2018)	C	Participants indicate whether a presented letter is the same as or different from the letter presented prior to the immediately preceding one.	Reaction time across all 2-back trials.
**Switching**			
Task Switching (Pashler et al., 2001)	C	Participants must rapidly decide whether a number is high or low (one task set), or odd or even (another task set), depending on the color of the screen.	Reaction time-based global task-switch cost (difference in reaction time between non-switch trials in a two-task set block and single-task set block).
DKEFS Trail Making (Salthouse, 2011)	W	In condition B of this trail making test (used in this analysis), the participant draws lines to connect circled numbers and letters in an alternating numeric and alphabetic sequence (i.e., 1-A-2-B, etc.) as rapidly as possible.	Time on Trails B. This condition shows the highest sensitivity to aging.
**Inhibition**			
Flanker (Papadopoulos et al., 2015)	C	The stimulus is a row of five horizontal arrows, and the participant’s task is to report the direction (left or right) in which the center target arrow points by pressing one key for left and a different key for right. The flankers are the two arrows on each side. In the congruent condition, they point in the same direction as the central arrow (<<<<< or >>>>>). In the incongruent condition, they point in the opposite direction as the target arrow (>><>>, <<><<)	Reaction time for incongruent condition. This condition is particularly sensitive to response efficiency.
**Generalized (updating, shifting, and inhibition)**			
Digit Symbol Substitution (Jaeger, 2018)	W	Participants match symbols to numbers referencing a key located at the top of the page.	The number of correct symbols within the allowed time (120 s).
Means-End Problem-Solving (Vandermorris et al., 2013)	W	Participants read story stems in which a problem is stated at the beginning and an outcome at the end. The task is to fill in events that might have taken place between the discovery of the problem and its eventual solution. Participants are given two stories during each untimed test.	Number of relevant steps to reach solution

### Analyses

#### Hypothesis 1: brain modularity increases attributed to the intervention

For each participant and for each intervention time point (pre-and post-intervention), we used the 264 regions (“nodes”) from Power et al. (2011) to generate resting state functional-connectivity matrices. The nodes will henceforth be referred to as “Power nodes.”

For the modularity analyses, we used a generalization of the Louvain algorithm (GenLouvain, Blondel et al., 2008; Jutla et al., 2011) to obtain communities from the functional-connectivity matrices of each subject (at each time point, pre- and post-intervention) while varying the structural resolution parameter gamma from 0.5 to 3 in increments of 0.5. Gamma is an optimization parameter that scales the relative importance of observed connections between nodes within a community and the weights of those connections expected under a random null model. Here, we used a null model in nodes to have the same number of connections (their degree), but where those connections were made randomly.

At each gamma level, the GenLouvain algorithm generates two output variables. One output refers to the community that each “Power node” is assigned to; a community is defined as a sub-network of nodes that are more tightly connected to each other than expected by chance. Mathematically, community assignment is represented by the variable “Ci” where “i” indexes the “Power node” that belongs to a community “C.” The second output is “modularity,” which measures the quality of the complete set of communities, known as a partition (Newman and Girvan, 2004; Reichardt and Bornholdt, 2006; Fasino and Tudisco, 2014). Mathematically, modularity is represented by the variable “Q.”

Although fast and accurate, the GenLouvain algorithm is stochastic (the Ci and related *Q*-value can vary from run to run). To account for this, we ran the algorithm 100 times. It generated 100 sets of community assignments. For each run of the algorithm, the median *Q*-value was recorded. This process of generating the median *Q*-value was conducted for every gamma-threshold parameter. Following this step, we tested for Group × Time interaction effects in the median *Q*-value across different gamma-thresholds. We evaluated these effects using a generalized linear mixed model (GLMMs; see Lo and Andrews, 2015) with maximal random effects estimation (Barr, 2013). Our final model was represented as median Q ∼ Group + Time + Group*Time + random intercept for participant. We used a “Gaussian” distribution with log link (a log link exponentiates the predictors in the model). Analyses were completed in R studio (version 1.1.4, package “lme4,” function glmer). See here for the detailed analyses.^2^

#### Hypothesis 2: post-executive function scores are representative of the acting group

A partial least squares regression-discriminant analysis (PLS-DA, Barker and Rayens, 2003) approach was used to examine the role of post-test scores on the tasks in group discrimination adjusted for pre-timepoint contributions of these same scores in the absence of intervention effects (i.e., the absence of effects due to active experiencing). Specifically, for each task, we derived the beta estimates of the post-on-pre-regression model for the active control condition. From these beta estimates, we got residuals for both the acting intervention group and the control group. In this manner, we were able to obtain a realization of the unobserved outcome for people in the acting intervention had they been assigned to the active control condition. These residuals were studentized and then included in the PLS-DA as predictors.

There are several advantages of using PLS-DA for the current study. First, it relates the predictor variables (i.e., the studentized task scores) to group membership (i.e., intervention or control group) as well as models the common covariance structure between all (studentized) task scores and class membership. This is achieved by finding a set of latent variables that describe maximum covariance between the task scores and group data. Relatedly, and second, PLS-DA is well-suited when there are likely to be moderate-to-strong correlations among the predictor variables, as is typically the case with executive functioning tasks. Third, it has been suggested that there is less value in isolating executive function subcomponents (i.e., updating, shifting, and inhibition) in neuropsychological analyses, particularly in the case of older adults (McAlister and Schmitter-Edgecombe, 2016). Thus, PLS-DA allowed us to model the joint contribution of all executive function subcomponents, rather than evaluating them separately. In our prior report, we examined control-adjusted gains using a latent change score model for each cognitive domain (Banducci et al., 2017). Here we apply a PLS-DA framework to simultaneously examine correlated indices of executive function (consistent with the operationalization of this construct by Miyake et al., 2000) and brain modularity, specifically, to differentiate groups. Our primary performance measure of executive function in the present study was reaction time, given greater inter- and intra-variability in this measure, compared to accuracy. Any bias due to factors such as processing speed would be accounted for by the studentization of scores, since studentization was done relative to the control group (controlling for processing speed and other confounds between groups) and involved normalization by standard deviation (controlling for processing speed and other confounds within individuals).

A list of all tasks with a Variable Importance in Projection (VIP) >1 were deemed as “discriminant tasks.” A VIP is used to summarize the contribution a measure makes to the PLS-DA model, based on the percentage of the explained residual sum of squares. Analyses were completed in R studio (version 1.2.5042; plsda function from the mixOmics package).

#### Hypothesis 3: post-intervention performance on discriminant tasks positively interacts with brain modularity to predict assignment to acting group

We developed separate logistic regression models to evaluate whether scores on each discriminant task determined group membership to acting, based on the contribution of modularity. Analyses were completed in R studio (version 1.2.5042; glm function from the stats package).

## Results

**FIGURE 1:**
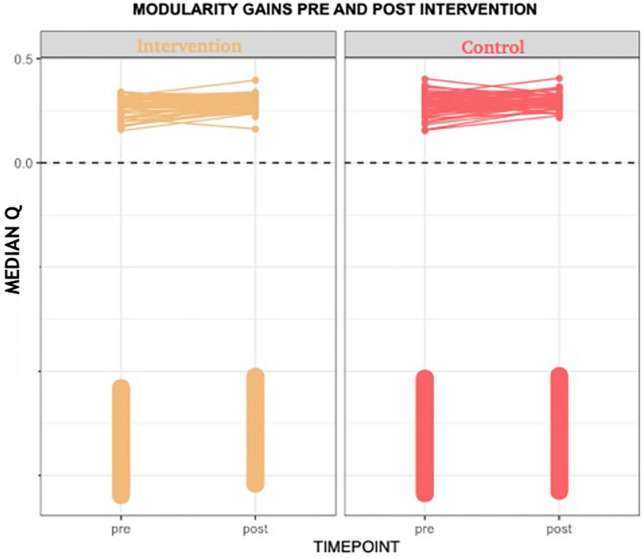
Median Q (modularity) pre- and post-intervention. Using a mixed-model analysis, we found an increase in modularity in the intervention group, relative to controls and the pre-intervention timepoint (estimate: 0.04, *p*-value = 0.01 at a significance level alpha ≤0.05).

Consistent with our first hypothesis, our mixed-model analysis suggested a significant increase in modularity in the intervention group, relative to controls and the pre-intervention timepoint ($\hat{\beta }$ estimate: 0.04, *p*-value = 0.01 at the significance level α0.05; see Figure 1).

**FIGURE 2:**
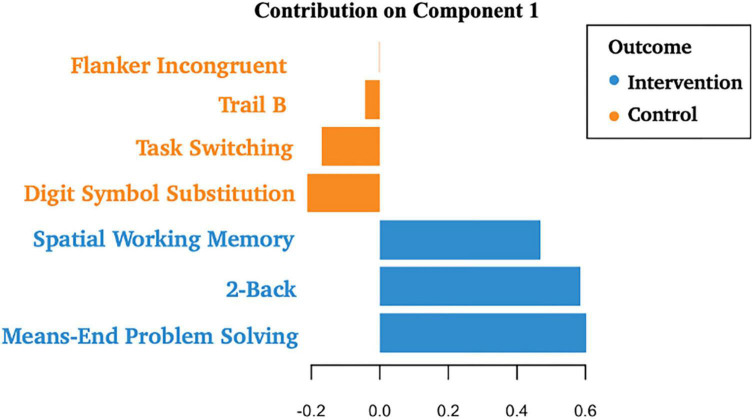
Component loadings for cognitive tasks. A partial least squares regression-discriminant analysis (PLS-DA) approach was used to examine the role of post-test cognitive scores in group discrimination adjusted for pre-timepoint contributions of these same scores in the absence of intervention effects. These results suggest that post-test scores in Spatial Working Memory, 2-Back, and Means-End Problem Solving were more likely to be associated with the intervention group, not the control group.

Descriptive statistics related to the tasks are presented in Table 2. The PLS-DA analysis showed that three tasks were found to carry group-separating information (Variable Importance in Projection >1). These discriminant tasks were Spatial Working Memory, 2-back, and Means-End Problem-Solving. Component loadings on these task measures suggest that post-intervention reaction time performance in these tasks were representative of the intervention group, not the control group. The amount of variance explained by this model was 19%. The error rate was 36%. Please see Table 3 and Figure 2 for the related results.

**TABLE 2:** Descriptive statistics for tasks.

Task (measure)	Time	Statistic	Acting	Control	Boxplot	*T*-value (*p*-value)
Spatial Working Memory (RT)	Pre	N	83	81	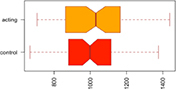	−0.83 (0.41)
		Mean (SD)	1,021.4 (183.7)	998.9 (163.2)		
	Post	N	81	79	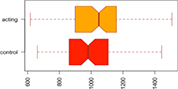	−1.55 (0.12)
		Mean (SD)	1,036.4 (187.2)	992.5 (171.8)		
2-Back (RT)	Pre	N	84	81	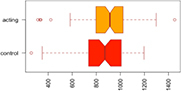	−1.68 (0.09)
		Mean (SD)	911.5 (199.6)	859.5 (197.0)		
	Post	N	81	79	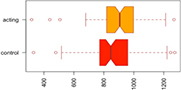	−1.69 (0.09)
		Mean (SD)	902.6 (162.7)	858.2 (169.2)		
Task Switch (RT)	Pre	N	83	77	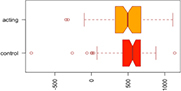	0.59 (0.55)
		Mean (SD)	478.6 (271.8)	504.5 (280.3)		
	Post	N	75	77	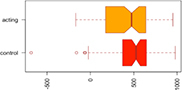	1.34 (0.18)
		Mean (SD)	406.2 (283.6)	467.7 (282.4)		
DKEFS Trail B (Time)	Pre	N	84	80	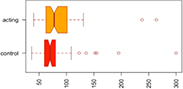	−1.66 (0.10)
		Mean (SD)	85.99 (35.1)	76.68 (36.6)		
	Post	N	75	77	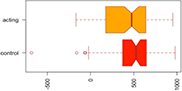	−0.84 (0.40)
		Mean (SD)	79.2 (33.6)	75.0 (29.1)		
Flanker Incongruent (RT)	Pre	N	75	73	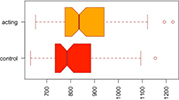	−**2.76 (0.01)**
		Mean (SD)	866.6 (124.7)	813.0 (111.7)		
	Post	N	79	73	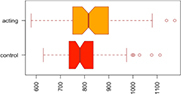	−**2.63 (0.01)**
		Mean (SD)	838.8 (119.1)	792.1 (99.1)		
Digit Symbol Subst. (Number of correct symbols)	Pre	N	83	81	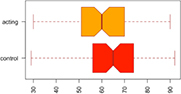	1.78 (0.08)
		Mean (SD)	60.9 (13.3)	64.5 (12.8)		
	Post	N	81	80	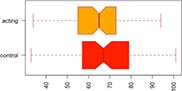	1.89 (0.06)
		Mean (SD)	64.0 (13.4)	68.1 (14.2)		
Means-End Problem Solving (Number of effective solutions)	Pre	N	84	81	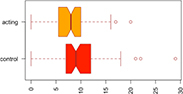	**2.06 (0.04)**
		Mean (SD)	8.4 (4.0)	9.8 (4.9)		
	Post	N	77	80	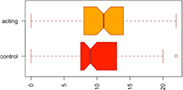	−1.42 (0.16)
		Mean (SD)	11.3 (4.6)	10.3 (4.3)		

*T*-values are based on two-sample *t*-tests of *mean(acting)-mean(control)* evaluated separately for pre- and post- timepoints. *P*-values in bold suggest significant difference in the means of the two groups.

**TABLE 3:** Loadings on projected components.

Variable	Loading	VIP*
**Spatial Working Memory**	**0.47**	**1.24**
**2-Back**	**0.58**	**1.55**
Task Switching	−0.17	0.45
Trails B	−0.04	0.12
Flanker Incongruent	0.00	0.00
Digital Symbol Substitution	−0.21	0.56
**Means-End Problem-Solving**	**0.60**	**1.59**

VIP*, Variable Importance in Projection. All tasks with VIP >1 are in bold. These results suggest that post-test scores in Spatial Working Memory, 2-Back, and Means-End Problem Solving were representative of the intervention group, not the control group. The amount of variance explained by this model is 19%. The error rate is 36%.

Based on the logistic regression analysis, we did not find any evidence of an interaction effect for modularity with any of the task performance scores. The related logistic regression results are presented in Table 4.

**TABLE 4:** Logistic regression models for the interaction between modularity (Q) and discriminant task performance scores.

Model	Interaction estimate (*p*-value)
Q + Spatial Working Memory + Q × Spatial Working Memory	−0.25 (0.23)
Q + 2-back + Q × 2-back	0.12 (0.70)
Q + Means-End Problem-Solving + Q × Means-End Problem-Solving	0.20 (0.43)

These results suggest that none of the interactions between modularity (q) and discriminant task scores were significant at a significance level α≤0.05. All estimates are relative to the control group.

## Discussion

Prior research has suggested that theater acting can facilitate improvements in cognitive performance. We designed a 4-week theater-acting intervention in older adults to characterize the cognitive and brain changes associated with such an intervention.

Our mixed model analysis revealed greater increase in modular organization in the AE intervention as compared to an active control, suggesting a possible conferral of flexible learning and optimization of brain functions (Sporns and Betzel, 2016).

Using a PLS-DA framework, we found a significant post-intervention contribution of tasks that uniquely indexed the updating component of executive function. These tasks were Spatial Working Memory, 2-back, and Means-End Problem-Solving, and they were representative of the intervention group (Table 3). In older adults, updating has been identified as a key predictor of lab-based assessments of functional status—defined as the ability to perform tasks of everyday living—including the ability to manage finances and medication (McAlister and Schmitter-Edgecombe, 2016; McDougall et al., 2019). Furthermore, updating as well as episodic memory processes appear to specifically benefit from broad-based engagement interventions that are targeted at this population (Park et al., 2014; Banducci et al., 2017; Myhre et al., 2017). Within this context, it is worth noting that the Means-End Problem Solving task captures both updating as well as episodic memory processes (Vandermorris et al., 2013). Integrating these points, it appears plausible that when contributions of all executive functioning tasks are considered, performance gains ascribed to acting are related to the updating aspects of executive function and potentially, episodic memory.

Using a logistic regression approach, we did not find any significant interactions between modularity and task performance that could clearly discriminate the two groups. Thus, across all analyses, our results suggest parallel increases in modularity and updating, but not interacting relationships in these two variables that could sufficiently differentiate group membership. Of interest, the only study that has directly demonstrated changes in modularity corresponding with changes in cognitive training, used an adaptive version of the n-back task (Finc et al., 2020). However, this study did not determine how groups differed based on the interaction between modularity and adaptive n-back performance. Within the experimental group, the authors found that an increase of modularity observed during the 2-back condition of this task was not correlated with behavioral performance after training. They theorized that an increase in modular organization reflected a general consequence of training and may not interact with behavioral improvement. Our study provided evidence in support of this speculation. That said, we did not use the adaptive version of the n-back task, which may better index flexible learning, and therefore, modularity.

There is considerable evidence that brain modularity, as well as the updating component of executive function, and episodic memory, are particularly sensitive to and decline with the aging process (McAlister and Schmitter-Edgecombe, 2016; Sporns and Betzel, 2016; Banducci et al., 2017; Maldonado et al., 2020). Our results suggest that an active experiencing acting intervention can facilitate improvements in brain-wide organization and these aspects of higher-order cognitive functions.

We recognize that there are limitations to our study. From a neuroimaging standpoint, our instruction to participants to keep their eyes closed may have led to their sleepiness, which we did not explicitly follow-up on. Our instruction was guided by the observation that the resting state scan was sandwiched between two visually demanding scanner tasks (tasks not presented in this study). Notwithstanding, we note that all participants were alert and responsive to instructions that followed immediately after the scan and their performance on the task that followed did not indicate drowsiness.

From a behavioral methods standpoint, we found an error rate of 36% when classifying both groups using PLS-DA. This error rate may signal reduced sensitivity to executive function measures due to education effects (McAlister and Schmitter-Edgecombe, 2016), as our sample was highly educated. We also lacked a diversity of executive function tasks that uniquely mapped onto each subcomponent as well as other higher order functions. For instance, the Wisconsin Card Sorting Test measures the ability for flexible learning—a characteristic of executive function and modularity—but was not included in our battery of tests.

We acknowledge that the time of day when cognitive tests and MRI scans were conducted may have exerted an influence on the magnitude of performance on executive function tasks as well as whole-brain modularity measures (Schmidt et al., 2007; Farahani et al., 2022). We did not explicitly track participants’ habitual sleep-wake patterns to account for such individual-level presentations. Nonetheless, we surmise that the random assignment of participants into the two groups may control for time-of-day effects. Future studies should be explicit in their declaration of testing and scanning times.

We also note that while both groups were designed to be internally rewarding, we did not explicitly measure participants’ motivation, which can affect behavioral engagement. Thus, we cannot definitively say the extent to which our results are influenced by group differences in motivation.

Finally, the acting-theater researchers who supervised the two groups were also co-authors of this study (TN and HN) and the extent to which this factor may have influenced the research outcomes is difficult to determine.

## Conclusion

We conducted a 4-week active experiencing-based acting intervention in older adults, within a randomized control trial framework. We anticipated enhancements in modularity and executive functioning, attributed to the intervention. We also investigated how changes in modularity were related to identified changes in executive function. These analyses were primarily conducted in a discriminant analysis framework. We found that the intervention group was characterized by higher post-intervention scores on tasks indexing the updating component of executive function. These tasks were termed “discriminant tasks.” None of the discriminant tasks significantly interacted with change in modularity to predict group assignment (i.e., intervention group or control group). We suggest that some attributes of modularity—such as flexible learning—were not effectively captured by our executive function measures. Nonetheless, our findings are promising since they suggest that an active experiencing based acting intervention can facilitate improvements in aspects of higher-order cognitive functions and brain-wide organization.

## Data availability statement

The datasets presented in this study can be found in online repositories. The names of the repository/repositories and accession number(s) can be found below: https://osf.io/pr4kt/?view_only=2caec53f73df48178fe0b53d1d19a003, identifier: DOI 10.17605/OSF.IO/PR4KT.

## Ethics statement

The studies involving human participants were reviewed and approved by the University of Illinois Institutional Review Board. The patients/participants provided their written informed consent to participate in this study.

## Author contributions

TN, HN, and AK designed and directed the project. AR conceived of the presented idea and developed the conceptual framework. RB assisted in performing all analyses. AD aided in interpreting the results. PB and MV verified the analytical methods. All authors provided critical feedback and helped shape the research, analysis, and manuscript.

## Footnotes

1
www.fmrib.ox.ac.uk/fsl

2
https://rpubs.com/saraswatiSattva/407764
